# Fenofibrate: A Nonlithogenic Means of Recurrent Drug-Induced Pancreatitis

**DOI:** 10.1155/2018/4580860

**Published:** 2018-09-09

**Authors:** Thamer Kassim, Joy-Marie Hermes, Abdullah Abdussalam, Ahmed Aly, Subhash Chandra

**Affiliations:** ^1^Department of Medicine, Division of General Internal Medicine, Creighton University, Omaha, NE, USA; ^2^Creighton University School of Medicine, Omaha, NE, USA; ^3^Department of Medicine, Division of Gastroenterology, Creighton University, Omaha, NE, USA; ^4^Department of Radiology, Creighton University, Omaha, NE, USA

## Abstract

Medications account for a small portion of the various etiologies of acute pancreatitis. Prompt identification of drugs as the inciting factor decreases disease recurrence and unnecessary invasive diagnostic intervention. This case is a report of fenofibrate-induced acute pancreatitis including a disease recurrence with continuation of fenofibrate which subsequently resolved after drug discontinuation. The patient underwent invasive diagnostic evaluation including endoscopic ultrasound with fine needle aspiration and endoscopic retrograde cholangiopancreatography (ERCP). Based on exclusion of other disease etiologies and a positive drug rechallenge, fenofibrate fits as a class 1A medication in the classification of drug-induced pancreatitis.

## 1. Introduction

The etiology of acute pancreatitis is identified in 70-90% of cases. Gallstones (up to 45%) and alcohol use (up to 35%) overwhelmingly produce most cases. Other precipitating factors include hypertriglyceridemia, ERCP, hypercalcemia, smoking, scorpion venom, malignancy, infection, and trauma (10% combined) [1]. The remaining 10-30% of cases are classified as idiopathic pancreatitis [1, 2]. This percentage continues to shrink with improvement of diagnostic modalities and increased literature regarding drug-induced pancreatitis, which accounts for approximately 2% of cases [2, 3].

Drug-induced pancreatitis is a diagnosis fraught with difficulty. It is made when symptoms resolve with drug cessation and recur in accordance with a rechallenge of the same drug [4]. In this report, we examine a case of fenofibrate-induced recurrent pancreatitis.

## 2. Case Presentation

**Figure 1:**
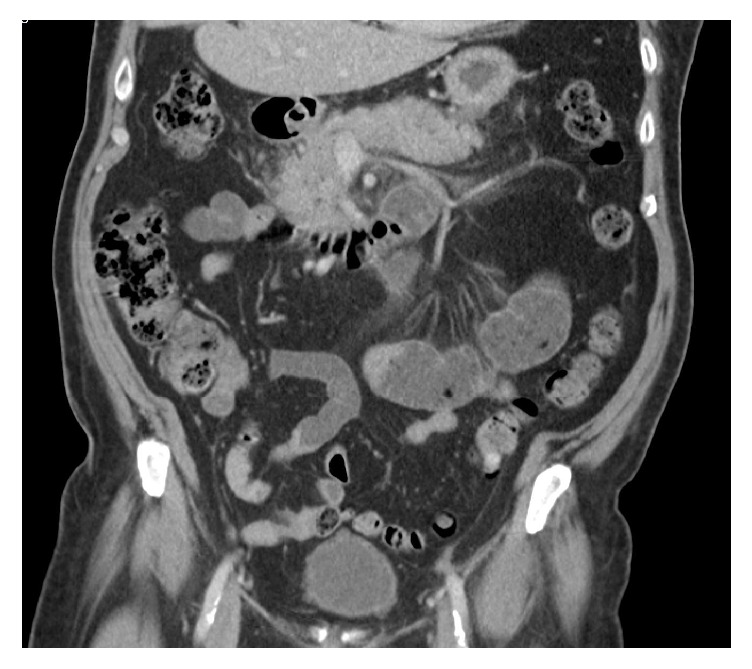
Coronal contrast enhanced CT image showing a homogenously enhancing bulky pancreas with peripancreatic haziness (arrows) with no evidence of collections consistent with noncomplicated acute interstitial pancreatitis.

A 79-year-old male with a medical history of cholelithiasis, for which he underwent cholecystectomy 11 years ago, hyperlipidemia, essential hypertension, and paroxysmal atrial fibrillation was admitted for mild acute pancreatitis. His presentation included severe epigastric pain radiating to the back, lipase of 1840 u/L, and no organ failure. He denied fever, chills, or body aches. The patient denied alcohol consumption and reported to have quit smoking 41 years ago. Liver chemistries, serum bilirubin, serum triglycerides, and immunoglobulin subclasses were within normal limits (Table 1). Abdominal CT scan demonstrated peripancreatic haziness consistent with noncomplicated acute pancreatitis (Figure 1). Home medications included atorvastatin for several years and the addition of 160 mg fenofibrate six months prior to admission. These medications were held upon admission but resumed at discharge. Full medication lists on admission and at discharge are shown in (Table 2). The patient was managed with fluid resuscitation and pain control. The patient recovered well and was able to tolerate regular diet without any pain or nausea. The patient was discharged in a stable condition after three hospital admission days, and lipase level at the day of discharge was 307 u/l.

**Figure 2:**
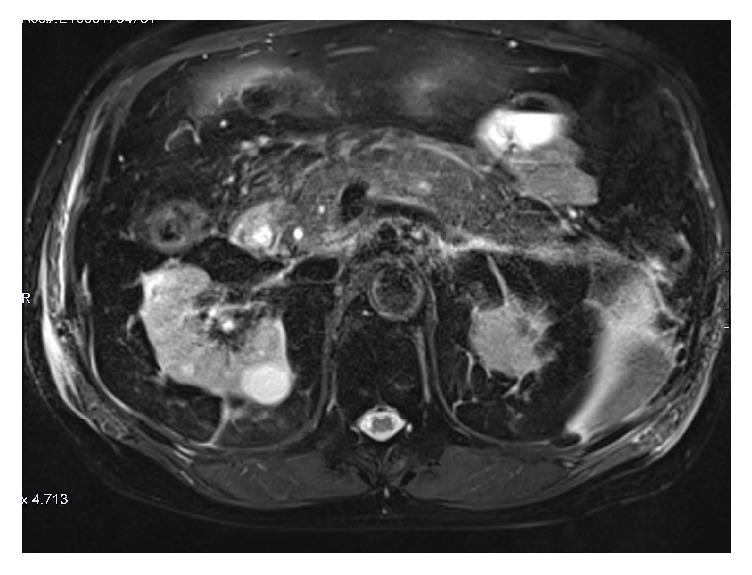
Fat suppressed T2W axial image showing both peripancreatic haziness and edema (arrows) consistent with acute interstitial pancreatitis.

Three days later, the patient returned with similar symptoms. Lipase levels were >30,000 u/l (Table 1), and magnetic resonance cholangiopancreatography showed acute interstitial edematous pancreatitis (Figure 2). The patient was treated conservatively with intravenous fluid resuscitation, pain control, and nothing per mouth until his symptoms resolved. Four days after his second admission, the patient recovered well and was discharged home in a stable condition. Fenofibrate and atorvastatin were discontinued (Table 2).

Two months later, the patient remained asymptomatic and returned for further workup to rule out an alternative cause to fenofibrate-induced pancreatitis. Endoscopic ultrasound (EUS) was done, which showed a 12 mm x 20 mm pancreatic head mass without pancreatic duct stenosis, strictures, or dilation. There were no common bile duct abnormalities. Fine needle aspiration was performed, and cytology was negative for malignant cells. Endoscopic retrograde cholangiopancreatography (ERCP) was performed to place a prophylactic pancreatic duct stent. Cholangiogram revealed a normal biliary system without dilation or strictures. The biliary tree was swept, and nothing was found. A prophylactic pancreatic duct stent was placed and a prophylactic sphincterotomy was performed. No recurrence of pancreatitis has occurred as of 6-month follow-up.

Given the course of the patient's illnesses in relation to fenofibrate usage, the timespan of drug initiation, and the fact that he had been taking atorvastatin for many years without previous signs or symptoms of acute pancreatitis, we hypothesize that his recurrent disease is probably due to the use of fenofibrate.

## 3. Discussion

Drug-induced pancreatitis accounts for 0.1%-2% of all cases of acute pancreatitis. Diagnosis of drug-induced pancreatitis is made when symptoms resolve with drug cessation and recur in accordance with a rechallenge of the same drug [4]. The disease is classified, from class I to IV, based on the number of cases reported, demonstration of a consistent latency, and reaction with rechallenge [5]. Three patterns of consistent latency are noted: a short latency of less than 24 hours, an intermediate latency of 24 hours to 30 days, and a long latency of greater than 30 days [5].

Typically, severity of drug-induced pancreatitis ranges from mild to moderate, but severe and even fatal cases may occur. The mechanism behind the condition remains controversial and varies between different offending medications [4]. Possible mechanisms include pancreatic duct constriction, cytotoxic effects, hypersensitivity reactions, and accumulation of toxic metabolites. Some drugs may cause pancreatitis indirectly by inducing hyperlipidemia or hypercalcemia [4, 6].

Lipid-modifying treatment with fibrates has been associated with the development of acute pancreatitis [7, 8]. According to the Coronary Drug Research Project, clofibrate was associated with a 50% increased risk of developing cholelithiasis or cholecystitis versus placebo, thus increasing the risk of acute pancreatitis [7]. Fenofibrate is believed to be less likely to induce gallstone formation while bezafibrate may raise biliary cholesterol concentration, thereby increasing the potential for gallstone formation and possibly resulting in acute pancreatitis [8]. However, there is limited data to support this theory.

Prior case reports have implicated clofibrate and bezafibrate as potential precipitating causes of acute pancreatitis [9, 10]. The only case involving fenofibrate-associated pancreatitis was reported by McDonald et al. [1] (2002) in which a patient on simvastatin, a class 1A medication, and fenofibrate developed acute pancreatitis. In their report, there was no rechallenge as the patient expired during a complicated hospital course.

Our patient was on atorvastatin, a class III medication, for many years without complications. He developed acute pancreatitis subsequent to the addition of fenofibrate six months prior to his initial presentation. The patient developed a recurrence of pancreatitis within 72 hours of fenofibrate rechallenge. In addition, the patient had a prior cholecystectomy and an EUS was negative for biliary stones, strictures, dilations, sludge, and chronic pancreatitis. A 12 mm x 20 mm pancreatic mass was found without pancreatic duct stenosis, strictures, or dilation and FNA revealed no malignant cells. Given that all other known causes of acute pancreatitis were ruled out, along with a positive rechallenge to the medication, we theorize that fenofibrate was a probable cause of recurrent drug-induced pancreatitis. Also, it is possible that the combination of fenofibrate and statin use can increase the risk for the disease. Furthermore, we believe that fenofibrate may produce pancreatitis by a different mechanism than previously theorized in literature, as there was no evidence of biliary stones or sludge in this case.

## 4. Conclusion

This case identifies fenofibrate as a probable cause of drug-induced pancreatitis, in addition to highlighting the importance of medication review in patients presenting with recurrent disease. We hypothesize that the mechanism of action regarding fenofibrate-induced pancreatitis is separate from the previously stated mechanism of lithogenic potential of other lipid lowering agents. It is also possible that the combination of fenofibrate with a statin can increase the risk of recurrent drug-induced pancreatitis.

## Figures and Tables

**Table 1:** Laboratory markers: initial admission; second admission after drug rechallenge.

**Laboratory test**	**Initial Admission**		**Second admission - 72 hours after discharge**	**Reference range**
**Pancreatic enzymes**				
Amylase (u/l)	158		671	20 - 90
Lipase (u/l)	1840	307 at discharge	>30,000	73-393

**Basic metabolic panel**				
Glucose (mg/dl)	101		75	70 - 100
Sodium (mmol/L)	136		138	135 - 145
Potassium (mmol/L)	4.2		3.6	3.7 - 5.1
Chloride (mmol/L)	104		102	96 - 110
Carbon dioxide (mmol/L)	24.0		28	22.0 - 32.0
Calcium (mg/dl)	9.0		8.8	8.5 - 10.5
Creatinine (mg/dl)	1.46		1.36	0.60 - 1.30
Blood urea nitrogen mg/dl	22		17	6 - 24
Glomerular filtration rate (mL/min/1.73 m2)	45		49	>=90
Anion Gap (mmol/L)	12		12	<=20

**Hepatic function panel**				
Alanine aminotransferase (u/l)	28		44	12 - 78
Aspartate aminotransferase (u/l)	33		37	10 - 40
Alkaline Phosphatase (u/l)	61		86	33 - 138
Total bilirubin (mg/dl)	1.1		0.5	0.0 - 1.5
Total protein (gm/dl)	7.5		7.4	6.0 - 8.4
Albumin (gm/dl)	3.4		3.3	3.5 - 5.0

**Immunoglobulin subclasses**				
IgG Subclass 1 (mg/dl)	626			405 - 1011
IgG Subclass 2 (mg/dl)	498			169 - 786
IgG Subclass 3 (mg/dl)	33			11 - 85
IgG Subclass 4 (mg/dl)	19			3 - 201

**Other laboratory tests**				
Triglycerides (mg/dl)	74			<149
White blood cell (k/ul)	14.7		10.8	4.0-12.0
Hemoglobin (gm/dl)	15.2		14.1	13.5-17.5
Platelets (k/ul)	237		298	140-440

**Table 2:** Complete medication list prior to each admission and on discharge.

**On first admission**	**At first discharge**	**On second admission**	**At second discharge**
Atorvastatin 20 mg ever night	Atorvastatin 20 mg ever night	Atorvastatin 20 mg ever night	Allopurinol 300 mg daily

Fenofibrate 160 mg every night	Fenofibrate 160 mg every night	Fenofibrate 160 mg every night	Acetaminophen 500 mg every 6 hours PRN

Allopurinol 300 mg daily	Allopurinol 300 mg daily	Allopurinol 300 mg daily	Lisinopril 10 mg daily

Acetaminophen 500 mg every 6 hours PRN	Acetaminophen 500 mg every 6 hours PRN	Acetaminophen 500 mg every 6 hours PRN	Warfarin 5 mg daily

Lisinopril 10 mg daily	Lisinopril 10 mg daily	Lisinopril 10 mg daily	Propafenone 225 mg daily

Warfarin 5 mg daily	Warfarin 5 mg daily	Warfarin 5 mg daily	Fluticasone 2 sprays in each nostril every night

Propafenone 225 mg daily	Propafenone 225 mg daily	Propafenone 225 mg daily	Multivitamin (centrum silver) every day

Fluticasone 2 sprays in each nostril every night	Fluticasone 2 sprays in each nostril every night	Fluticasone 2 sprays in each nostril every night	Vitamin C (ascorbic acid) 1000 mg every day

Multivitamin (centrum silver) every day	Multivitamin (centrum silver) every day	Multivitamin (centrum silver) every day	

Vitamin C (ascorbic acid) 1000 mg every day	Vitamin C (ascorbic acid) 1000 mg every day	Vitamin C (ascorbic acid) 1000 mg every day	

Co-Q 100 mg every day	Co-Q 100 mg every day	Co-Q 100 mg every day	
